# Comparative Quantitative Profiling of Protein Lactylation Reveals a Dynamic Tissues-Specific Network Associated with Metabolic Specialization in Yaks

**DOI:** 10.3390/ani16142228

**Published:** 2026-07-18

**Authors:** Zhijuan Wu, Huan Yang, Junyu Chen, Jiabo Wang, Jikun Wang, Ming Zhang, Zhixin Chai

**Affiliations:** 1 College of Animal & Veterinary Sciences, Southwest Minzu University, Chengdu 610041, China; wzjdream2005@163.com (Z.W.);; 2 Key Laboratory of Qinghai-Tibetan Plateau Animal Genetic Resource Reservation and Utilization, Ministry of Education, Southwest Minzu University, Chengdu 610041, China; 3 Sichuan Qinghai-Tibet Plateau Herbivore Livestock Engineering Technology Center, Southwest Minzu University, Chengdu 610225, China

**Keywords:** yak, lactate, lactylation, metabolism

## Abstract

Protein lysine lactylation is an emerging post-translational modification with broad roles in metabolic regulation. The yak (*Bos grunniens*) has evolved strong metabolic adaptability on the Qinghai–Tibetan Plateau, yet its tissue-specific lactylation patterns remain poorly characterized. Here, we collected liver, muscle, and heart tissues from three adult male yaks (4.5 years; 305–355 kg) and integrated quantitative proteomics with lactylomics to map lactylation profiles across these tissues. After normalizing each lactylation site to its parent protein abundance and applying Benjamini–Hochberg correction, we identified 628, 982, and 541 differentially lactylated sites (|log2FC| ≥ 0.585, adjusted *p* < 0.05) in liver–muscle, liver–heart, and muscle–heart comparisons, with median fold changes of 4.11, 7.08, and 3.08, corresponding to 267, 372, and 219 proteins, respectively. Subcellular localization showed that approximately 27–30% of these sites were localized to mitochondria. Functional enrichment across these comparisons consistently highlighted pathways such as the TCA cycle (fold enrichment: 1.56–2.38) and HIF-1 signaling (up to 2.69). A total of 135 proteins were common to all three comparisons, some with both up- and downregulated sites within the same tissue pair. Our results reveal decoupling between protein abundance and lactylation levels, tissue-specific lactylation patterns on the same proteins, and expression-independent lactylation of key enzymes (e.g., LDHA, SIRT3). Functional enrichment suggests lactylation serves as a multimodal regulatory mechanism coordinating energy metabolism, protein homeostasis, and electromechanical coupling across tissues. Expression profiling of lactate-metabolizing enzymes and lactylation regulators further supports an organ-specific model of post-translational regulation. Collectively, these findings detail a complex, tissue-specific lactylation network in yaks and provide insights into the metabolic homeostasis essential for high-altitude life.

## Simple Summary

Yaks live on the Qinghai–Tibetan Plateau, a harsh environment with low oxygen, cold, strong sunlight, and little food. To survive, yaks have unique ways to manage their energy. One key process involves a substance called lactate, once seen only as a waste product, which can attach to proteins and change how they work—this is called “lactylation.” In this study, we wanted to see how lactylation patterns differ in three important organs: liver, muscle, and heart. We measured both the amount of each protein and the level of lactylation on them. Our results show that each organ has its own unique lactylation pattern. Importantly, the level of lactylation often did not match the amount of the protein itself, meaning that lactylation acts as an independent fine-tuning mechanism. Some proteins even showed opposite lactylation changes when comparing different organ pairs. This organ-specific control system likely helps yaks keep proteins working properly, maintain energy balance, and support proper heart and muscle function in harsh conditions. Understanding these natural regulatory patterns may provide insights for improving livestock breeding and for studying human diseases linked to low oxygen, such as certain heart and metabolic disorders.

## 1. Introduction

The Qinghai–Tibetan Plateau, characterized by extreme high-altitude conditions with hypobaric hypoxia, frigid temperatures, intense UV radiation, and nutrient-scarce vegetation [1,2], represents one of Earth’s most ecologically extreme environments. Within this habitat, the yak (*Bos grunniens*) has evolved as the dominant bovine species adapted to year-round grazing above 2500 m, with some populations seasonally ranging above 5000 m [3]. As a globally significant genetic resource and cornerstone of plateau pastoralism, yaks exhibit distinctive physiological adaptations in metabolic regulation and energy homeostasis [1,4,5]. Multidisciplinary exploration of these adaptations will illuminate evolutionary survival strategies and reveal their potential for applications in agricultural breeding and hypoxia-related biomedicine.

Anaerobic metabolism is an essential mechanism for yaks to acclimate to chronic high-altitude hypoxia while maintaining systemic homeostasis [5,6]. Through glycolysis, lactate dehydrogenase A (LDHA) catalyzes pyruvate-to-lactate conversion, yielding a molecule with dual roles beyond its classical identity as a metabolic end-product. First, lactate acts as an inter-organ, inter-cellular, and intra-cellular energy shuttle (e.g., muscle-to-heart, astrocyte-to-neuron, cytosol-to-mitochondria) [7]. Second, it serves as a precursor for protein lysine lactylation (Kla), a newly characterized post-translational modification (PTM) [8,9]. The landmark identification of histone-specific lactylation by Zhang et al. revealed lactate’s capacity for epigenetic regulation [8], later extended to non-histone proteins [10]. Through modifications of both histone and non-histone targets, lactylation modulates diverse biological processes, including development [11], neural function [12], inflammation [9], and cancer [13]. However, key questions remain regarding its spatiotemporal dynamics and downstream effectors in context-dependent pathways.

The dynamics of protein lactylation are governed by the opposing actions of lactyltransferases and delactylases, forming a precise “writer-eraser” regulatory system. Lactylation can be catalyzed by enzymes such as the histone acetyltransferase p300, which exhibits broad substrate specificity and can utilize lactyl-CoA [14,15]. More recently, the alanyl-tRNA synthetase AARS1 (cytosolic) and AARS2 (mitochondrial) have been identified as lactate sensors and lactyltransferases [16]. Conversely, the NAD^+^-dependent deacetylase sirtuin 3 (SIRT3) functions as a potent delactylase, efficiently removing these lactyl groups from both histones and non-histone proteins [17,18,19]. This coordinated regulation highlights lactylation as a dynamic and responsive signaling mechanism in metabolic adaptation. However, the tissue-specific expression and functional roles of these regulatory enzymes in yaks remain largely unexplored.

Yaks exhibit unique physiological adaptations to high-altitude environments, yet the underlying molecular mechanisms, particularly the role of PTMs like lactylation, remain incompletely understood. Research on metabolic enzyme PTMs in livestock significantly lags behind human studies, despite their critical roles in energy metabolism [20]. Recently, protein lactylation has emerged as a key mechanism for deciphering environmental adaptation [21,22]. However, existing studies in yaks have been primarily qualitative, leaving a gap in our understanding of the quantitative, tissue-specific dynamics of this modification. Specifically, it remains unclear whether lactylation levels differ quantitatively among metabolically active tissues, whether these differences are independent of protein abundance, and how they coordinate tissue-specific metabolic strategies under chronic hypoxia. The liver, skeletal muscle, and heart represent divergent metabolic phenotypes—gluconeogenesis/ketogenesis, glycolytic/thermogenic, and oxidative/contractile, respectively. We hypothesized that lactylation would be highest in the liver (reflecting high lactate flux) and show tissue-specific site occupancy patterns that align with these distinct metabolic demands. Building on our previous qualitative mapping of the lactylome in yak tissues [5], the aim of this study was to use quantitative lactylomics integrated with matched proteomics to correct for protein abundance biases, systematically compare lactylation patterns and differentially modified sites across the liver, muscle, and heart, and examine the expression of key enzymes involved in lactate metabolism and lactylation regulation. Through this approach, we sought to determine whether lactylation exhibits tissue-specific quantitative patterns that correlate with known metabolic phenotypes, and to provide a quantitative reference for future mechanistic studies.

## 2. Materials and Methods

### 2.1. Sample Preparation

Biological samples were collected from three mature male Jiulong yaks, located at an ecological altitude of 2885 m, with identical age and weight parameters (4.5 years; 305–355 kg) as those in our previous study [5]. All the animals were from the same herd, maintained under identical feeding and management conditions, and slaughtered at the same time of day. Following euthanasia performed in accordance with approved slaughter protocols, we immediately harvested heart, liver, and longissimus dorsi muscle tissues, which were flash-frozen in liquid nitrogen for subsequent biochemical analyses. All procedures were approved by the Institutional Animal Care and Use Committee of Southwest Minzu University (SMU-202501149) and the Regional Ethics Review Board for Animal Research, in compliance with international animal welfare standards.

The samples extracted from yak heart, muscle and liver tissues (*n* = 3 per tissue) were grinded with liquid nitrogen into cell powder and then transferred to a 5 mL centrifuge tube. After that, four volumes of lysis buffer (8 M urea, 1% protease inhibitor cocktail, 3 μM trichostatin A and 50 mM nicotinamide) was added to the cell powder, followed by sonication three times on ice using a high intensity ultrasonic processor (Scientz, Ningbo, China). The remaining debris was removed by centrifugation at 12,000× *g* at 4 °C for 10 min. Finally, the supernatant was collected and the protein concentration was determined with a BCA kit (Beyotime, Shanghai, China) according to the manufacturer’s instructions.

Equal protein amounts from each sample were enzymatically digested. Sample volumes were normalized with lysis buffer, and proteins were precipitated by the slow addition of trichloroacetic acid (TCA) to 20% (*w*/*v*) final concentration with vortex mixing, followed by incubation at 4 °C for 2 h. After centrifugation (4500× *g*, 5 min), the supernatant was discarded, and the pellets were washed 2–3 times with ice-cold acetone, then air-dried. The dried precipitates were resuspended in 200 mM triethylammonium bicarbonate (TEAB) and dispersed by sonication. Proteins were then reduced with 5 mM dithiothreitol (56 °C, 30 min) and alkylated with 11 mM iodoacetamide (room temperature, 15 min, in darkness). For digestion, trypsin was added at a 1:50 (*w*/*w*) enzyme-to-protein ratio and incubated overnight.

To enrich modified peptides, tryptic peptides dissolved in NETN buffer (100 mM NaCl, 1 mM EDTA, 50 mM Tris-HCl, 0.5% NP-40, pH 8.0) were incubated with pre-washed antibody beads (PTM BIO Cat# PTM1404, Hangzhou, China) at 4 °C overnight with gentle shaking. Then the beads were washed for four times with NETN buffer and twice with deionized water. The bound peptides were eluted from the beads with 0.1% TCA for three times. Finally, the eluted fractions were combined and vacuum-dried.

For proteome samples, peptides were desalted using a Strata X SPE column per the manufacturer’s protocol. For lactylome samples, peptides were desalted using C18 ZipTips (0.6 μL bed volume; Millipore, Burlington, MA, USA) per the manufacturer’s protocol. After desalting, peptides were dissolved in mobile phase A (0.1% formic acid, 2% acetonitrile in water). Separation was performed on a NanoElute UHPLC system (Bruker Daltonics, Bremen, Germany) equipped with a home-made reversed-phase analytical column (25 cm length, 100 μm i.d.). The mobile phase consisted of solvent A and solvent B (0.1% formic acid in acetonitrile). For the proteome samples, a 20 min linear gradient was applied: 9% to 24% B over 0–8 min, 24% to 35% B over 8–12 min, 35% to 80% B over 12–16 min, and 80% B maintained for 16–20 min (flow rate: 450 nL/min). For the lactylome samples, a 30 min gradient was used: 6% to 22% B (0–18 min), 22% to 30% B (18–24 min), 30% to 80% B (24–27 min), and maintained at 80% B (27–30 min) (flow rate: 450 nL/min). Eluted peptides were ionized via a capillary ESI source (1.75 kV) and analyzed on a timsTOF Pro in dia-PASEF mode. Full MS scans (100–1700 *m*/*z*) were followed by 10 PASEF-MS/MS scans (400–1200 *m*/*z*) with 25 *m*/*z* isolation windows and collision energy spread linearly ramped from 20 to 59 eV based on *m*/*z*.

### 2.2. Bioinformatics Analysis

Raw MS data were processed using Spectronaut (v17.0, Biognosys, Schlieren, Switzerland) with default settings against the *Bos mutus* proteome database (Bos_mutus_72004_PR_20221125.fasta; 18,553 entries) supplemented with common contaminants and decoy sequences. Search parameters specified Trypsin/P digestion (≤2 missed cleavages), fixed carbamidomethylation (C), and variable modifications (methionine oxidation, N-terminal acetylation, lysine lactylation). Identification thresholds were set at 1% FDR for proteins, peptides, and PSMs. Additional filters required: (1) precursor and protein FDR ≤ 1%, (2) ≥1 unique peptide per protein, and (3) localization probability ≥ 0.75 for Kla sites.

To evaluate biological reproducibility, principal component analysis (PCA) was performed separately on the normalized proteomic and lactylomic datasets using the prcomp function in R (v4.3.0). Sample-to-sample Pearson correlation coefficients were calculated within each dataset to assess inter-individual consistency among the three biological replicates of each tissue.

Relative abundances of PTM sites were derived from peptide intensity values using Spectronaut (v17.0). No missing value imputation was applied. Undetected peptides/proteins (NaN or FALSE in Spectronaut) and non-specific peptides were excluded. Lactylation sites with localization probability > 0.75 were retained, and intensities from multiple precursor ions were summed prior to normalization. Site-specific normalization was then performed by scaling each modification site’s intensity to its corresponding parent protein abundance, thereby decoupling PTM stoichiometry from protein expression variations. For multiple group comparisons among liver, heart, and muscle tissues, the normalized relative quantification values were log2-transformed prior to statistical analysis to satisfy the assumptions of normality and homogeneity of variances required for parametric testing. This transformation effectively stabilizes variance and improves the approximation to a normal distribution. To assess tissue-dependent effects while accounting for inter-individual variation in the paired design (three tissues per animal), we used a two-way ANOVA without interaction based on a Randomized Block Model, *Y_ij_* = *μ* + *Tissue_i_* + *Animal_j_* + *ε_ij_*, where Tissue (liver, muscle, heart) was the fixed effect and Animal was the blocking factor. This ANOVA served as a global exploratory test prior to pairwise comparisons. For pairwise comparisons, two-tailed *t*-tests were performed on log2-transformed values, and the resulting *p*-values were adjusted by the Benjamini–Hochberg method. Differentially modified sites were defined as those with |log2FC| ≥ 0.585 and adjusted *p* < 0.05 [23,24].

We conducted comprehensive functional characterization of differentially lactylated proteins through an integrated bioinformatics workflow, including GO and KEGG pathway annotation (eggnog-mapper v2.0 with eggNOG v5.0.2), protein domain analysis (PfamScan v1.6 against Pfam 33.1), subcellular localization prediction (WoLF PSORT v0.2), and orthologous group classification (COG/KOG via EggNOG). Functionally enriched terms (Fisher’s exact test, *p* < 0.05) were hierarchically clustered (Euclidean distance, average linkage) and visualized using pheatmap (v1.0.12). Protein–protein interaction (PPI) networks were constructed from STRING v11.5 (*Bos taurus* proteome; confidence score > 0.7) with the top 50 interactions displayed using networkD3.

### 2.3. Western Blot

Protein lysates from yak liver, muscle, and heart (*n* = 3 per tissue) were separated by 12% or 8% SDS-PAGE and transferred onto PVDF membranes. The membranes were blocked with 5% non-fat milk for 1 h at room temperature, then incubated overnight at 4 °C with primary antibodies diluted in TBST as follows: anti-L-lactyl-histone H3 (K56) rabbit mAb (PTM BIO Cat# PTM-1421RM; 1:1000), anti-L-lactyl-histone H2B (K120) rabbit pAb (PTM BIO Cat# PTM-1423; 1:2000), anti-LDHA/EP300/AARS1 rabbit pAb (ABclonal Cat# A1146/A13016/A15017; 1:2000, Wuhan, China), anti-AARS2/SIRT3 rabbit pAb (ABclonal Cat# A7826/A7307; 1:1000), anti-α-tubulin rabbit pAb (Affinity Biosciences Cat# AF7010; 1:10,000, Nanjing, China), anti-histone H3 mouse mAb (Sino Biological Cat# 100005-MM01; 1:10,000, Beijing, China). After three washes with TBST (10 min each), the membranes were incubated for 1 h at room temperature with HRP-conjugated goat anti-rabbit IgG (Invitrogen Cat# 31460; 1:10,000, Waltham, MA, USA) or anti-mouse IgG (Invitrogen Cat# C31430100; 1:10,000). Signals were detected using chemiluminescent HRP substrate (BIO-RAD ChemiDoC™, Hercules, CA, USA).

## 3. Results

### 3.1. Integrated Proteomic and Lactylomic Analysis of Yak Tissues

Quality control analysis of post-acquisition mass spectrometry data confirmed the robustness of the lactylome and proteome datasets from yak liver, muscle, and heart tissue types, supporting their suitability for subsequent analyses. Three biological replicates clustered tightly within each tissue type in PCA, with clear separation among liver, muscle, and heart samples (Supplementary Figure S1A,B). Pairwise Pearson correlation coefficients among replicates exceeded 0.86 for the proteomic data and 0.89 for the lactylomic data (Supplementary Figure S1C,D), indicating high inter-individual reproducibility. The relative standard deviation (RSD) values ranged from 20% to 40% for lactylated proteins and from 10% to 30% for non-modified proteins, consistent with the dynamic nature of lactylation regulation. Quantitative mass spectrometry analysis identified a total of 2307 high-confidence lysine-lactylated sites (FDR < 1%) mapped to 658 proteins across these three tissues, while concurrent unmodified proteomic analysis detected 3988 proteins. Peptide length distribution ranged predominantly between 7 and 20 residues, consistent with theoretical tryptic digestion patterns. Comparative analysis revealed markedly higher lactylation intensity in liver than in muscle or cardiac tissue, despite comparable total protein expression levels across tissues, suggesting tissue-specific lactylation regulation may correlate with metabolic activity.

**Figure 1:**
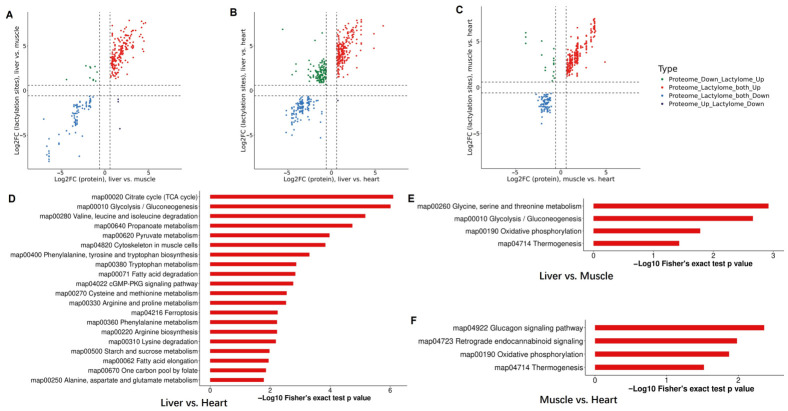
Integrated proteomic and lactylomic analysis of yak tissues. (**A**–**C**) Joint patterns of protein expression and lactylation levels (Quadrants: I, high protein/high lactylation; II, low protein/high lactylation; III, low protein/low lactylation; IV, high protein/low lactylation) for Liver vs. Muscle, Liver vs. Heart, and Muscle vs. Heart. Vertical/horizontal dashed lines mark log2FC = ±0.585 for protein abundance and lactylation intensity, respectively. Differential proteins and lactylation sites defined by |log2FC| ≥ 0.585 and adjusted *p* < 0.05 (Benjamini–Hochberg). (**D**–**F**) KEGG pathways enriched in proteins with discordant lactylation/expression (Quadrants II + IV) for the same three comparisons (Fisher’s exact test, *p* < 0.05).

Integrated analysis of proteomic and lactylomic data revealed distinct regulatory patterns through quadrant characterization (|log2FC| ≥ 0.585, adjusted *p* < 0.05; Figure 1A–C). Quadrant II (decreased protein expression with increased lactylation) suggested compensatory post-translational regulation, while Quadrant IV (increased expression with decreased lactylation) potentially indicated active suppression of lactylation to prevent overactivation (e.g., metabolic flux inhibition). Quadrants I and III demonstrated coordinated protein-lactylation changes.

KEGG enrichment analysis of Quadrants II and IV proteins (*p* < 0.05; Figure 1D–F, Supplementary Table S1) identified significantly enriched pathways involved in tissue-specific compensatory regulation. In Liver vs. Muscle (*p* < 0.05), enriched glycine/serine metabolism and glycolysis pathways indicated lactylation-mediated energy allocation, with oxidative phosphorylation and thermogenesis reflecting balanced metabolic demands. Liver vs. Heart analyses (27 total enriched pathways) highlighted the TCA cycle, pyruvate metabolism, and fatty acid degradation pathways, demonstrating optimized energy metabolism. Notably, cardiac adaptation relied on branched-chain amino acid metabolism, cytoskeleton remodeling, and dual signaling (HIF-1 for hypoxia adaptation; cGMP-PKG for tension control). In Muscle vs. Heart, enriched oxidative phosphorylation and thermogenesis pathways coexisted with glucagon and endocannabinoid signaling activation, implying potential hormonal modulation of cardiac energy metabolism. Collectively, these findings suggest lactylation may contribute to energy homeostasis through tissue-specific modulation: hepatic amino acid/lipid metabolism, muscular glycolytic-thermogenic activity, and cardiac functional adaptation involving multiple regulatory systems.

### 3.2. Comparative Analysis of Lactylation Sites and Proteins in Yak Tissues

**Figure 2:**
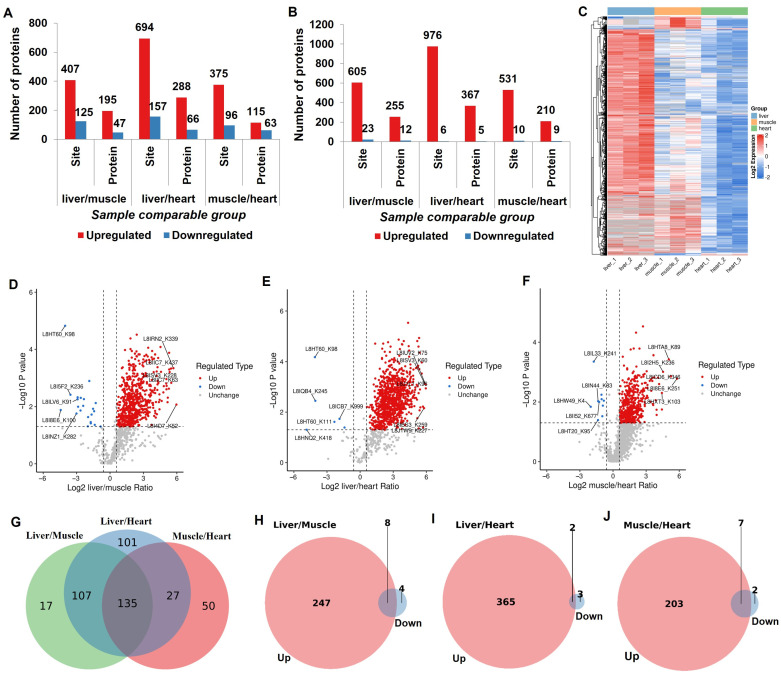
Comparative analysis of lactylation sites across yak tissues. (**A**) Differential lactylation sites (unadjusted). (**B**) Proteomic-normalized lactylation levels. (**C**) Heatmap clustering of significant sites (Z-score normalized). (**D**–**F**) Volcano plots: (**D**) Liver vs. Muscle, (**E**) Liver vs. Heart, (**F**) Muscle vs. Heart. (**G**) Shared lactylated proteins across comparisons. (**H**–**J**) Venn intersections of up/downregulated proteins: (**H**) Liver vs. Muscle (up [red], down [blue]), (**I**) Liver vs. Heart, (**J**) Muscle vs. Heart. Thresholds: |log2FC| ≥ 0.585, adjusted *p* < 0.05 (Benjamini–Hochberg correction). Vertical dashed lines in volcano plots (**D**–**F**) mark log2FC = ± 0.585, and horizontal dashed lines correspond to adjusted *p* = 0.05 (−log10*P* = 1.301).

Screening of differentially lactylated proteins across yak tissues revealed distinct modification patterns. Specifically, liver tissue exhibited 195 upregulated and 47 downregulated lactylated proteins compared to muscle tissue (|log2FC| ≥ 0.585, adjusted *p* < 0.05), and 288 upregulated and 66 downregulated proteins relative to heart tissue. Notably, the Muscle vs. Heart comparison displayed fewer differential modifications (115 upregulated/63 downregulated; Figure 2A). Following normalization against protein expression levels, the number of upregulated lactylated sites and their corresponding proteins increased, while the number of downregulated ones decreased. In the Liver vs. Muscle comparison, 628 differential lactylation sites were identified across 267 proteins; in Liver vs. Heart, 982 sites were mapped to 372 proteins; and in Muscle vs. Heart, 541 sites were found on 219 proteins (Figure 2B; Supplementary Table S2). This finding suggests that lactylation regulation may occur independently of protein abundance. Consistent with this, hierarchical clustering (Figure 2C) demonstrated robust tissue-specific patterning, with tight clustering of biological replicates.

Volcano plots identified tissue-specific, differentially modified Kla sites across three pairwise tissue comparisons (Figure 2D–F). Key examples include increased Kla at enolase (ENO1; L8ISV3; Sites: K5, K60, K80, K89, K103, K228, K256, K262, K330, K404, K418) and hemoglobin subunit beta (HBB; L8HT60; sites: K33, K35, K92) with decreased Kla at HBB (sites: K98, K111) and fructose-bisphosphate aldolase A (ALDOA; L8INZ1; sites: K94, K153, K282, K382) in Liver vs. Muscle. For Liver vs. Heart, elevated Kla occurred at ENO1 (sites: K5, K60, K80, K89, K103, K126, K193, K228, K256, K262, K330, K404, K418), HBB (sites: K33, K35, K92), ALDOA (sites: K160, K199, K364), and LDHA (L8ICD6; sites: K110, K184), while reductions were observed at HBB (sites: K98, K111). In Muscle vs. Heart comparisons, upregulated sites included ENO1 (sites: K89, K103, K126, K233, K330, K404, K418), LDHA (sites: K43, K110, K184, K261, K307, K346), HBB (sites: K35), and ALDOA (sites: K94, K151, K153, K160, K163, K199, K282, K364, K374, K382, K394), whereas ATP synthase subunits ATP5C1 (L8IN44; sites: K83) and ATP5H (L8HT20; sites: K95, K117) showed downregulation. Representative MS/MS spectra for ten selected lactylation sites distributed across the six proteins are presented in Supplementary Figure S2A–J, providing direct evidence for the modifications at these sites. Notably, although 135 differentially lactylated proteins overlapped across all comparisons (Figure 2G), their specific modification sites exhibited tissue-pair-dependent variation.

Furthermore, bidirectionally modified lactylated proteins (harboring both up- and downregulated sites) were identified: eight in Liver vs. Muscle, two in Liver vs. Heart, and seven in Muscle vs. Heart (Figure 2H–J). Notably, HBB was bidirectionally lactylated in both Liver vs. Muscle and Liver vs. Heart comparisons. Importantly, the specific sites upregulated and downregulated differed for each protein. Detailed lists of these proteins and their corresponding lactylated sites are provided in Supplementary Table S3.

PPI network analysis identified tissue-specific hubs among differentially lactylated proteins (Supplementary Figure S3). In Liver vs. Muscle, high-degree nodes (degree ≥ 35) included ATP synthase α/β/γ/d/O/b, succinate dehydrogenase (SDH), malate dehydrogenase (MDH), and cytochrome b-c1, suggesting potential hepatic energy allocation correlated with upregulated lactylation, while muscular lactylation may reduce SDH-mediated oxidation. In Liver vs. Heart, core interactors (degree ≥ 46; ATP synthase α/β/γ/d/b, succinyl-CoA ligase, cytochrome b-c1 Rieske) reveal elevated respiratory efficiency linked to Rieske lactylation in the heart and altered hepatic TCA flux associated with ketogenesis. For Muscle vs. Heart, hubs (degree ≥ 32; ATP synthase α/β/γ/d, DLD, MDH, SDH) indicate dihydrolipoyl dehydrogenase (DLD)-associated glycolytic–oxidative dynamics in muscle and maintained cardiac TCA integrity under hypoxia. Collectively, lactylation modifies core energy metabolism proteins in yaks, implicating potential roles in hypoxia adaptation.

### 3.3. Functional and Evolutionary Profiling of Differentially Lactylated Proteins Across Yak Tissues

Differentially lactylated sites across all three tissue comparisons were primarily localized to the cytoplasm (43.51–45.28%) and mitochondria (27.03–30.19%) (Supplementary Figure S4A–C). COG/KOG classification showed a consistent functional enrichment trend, albeit with varying degrees, across comparisons (Supplementary Figure S4D–F). The most significantly enriched categories were energy production and conversion and post-translational modification, protein turnover, and chaperones. Other notably enriched functions included Cytoskeleton and Signal Transduction Mechanisms; Intracellular Trafficking, Secretion, and Vesicular Transport; Lipid Transport and Metabolism; and Carbohydrate Transport and Metabolism. Notably, Secondary Metabolites Biosynthesis, Transport, and Catabolism and Cell Cycle Control, Cell Division, and Chromosome Partitioning were most strongly enriched in the Liver vs. Heart comparison. These results indicate that lactylation preferentially targets proteins central to core cellular processes, including energy metabolism, proteostasis, and structural organization.

**Figure 3:**
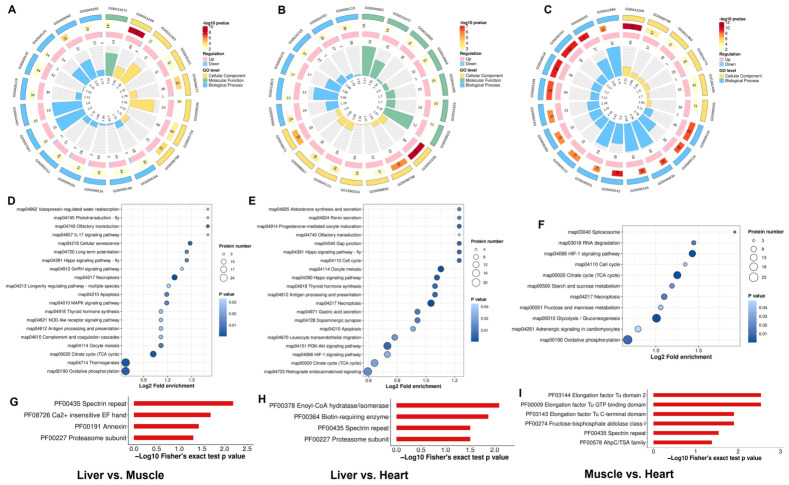
Functional landscape of differentially lactylated proteins in yak tissues. (**A**–**C**) Circos plots showing GO enrichment (BP/CC/MF) for tissue comparisons: (**A**) Liver vs. Muscle, (**B**) Liver vs. Heart, (**C**) Muscle vs. Heart. (**D**–**F**) Top 20 enriched KEGG pathways: (**D**) Liver vs. Muscle, (**E**) Liver vs. Heart, (**F**) Muscle vs. Heart. (**G**–**I**) Protein domain enrichment analysis: (**G**) Liver vs. Muscle, (**H**) Liver vs. Heart, (**I**) Muscle vs. Heart. All comparisons: *p* < 0.05 (Fisher’s exact test).

Functional enrichment analysis demonstrates that differentially lactylated proteins mediate environmental adaptation in yaks through multi-mechanistic coordination. Liver–muscle comparisons (Figure 3A,D,G) exhibited pronounced mitochondrial energy pathway enrichment (TCA cycle with a fold enrichment of 2.03, oxidative phosphorylation, thermogenesis), indicative of tissue-divergent ATP allocation-sustaining hepatic anabolism versus muscular thermogenesis. This metabolic framework was reinforced by: (i) proteostasis networks (proteasome subunits, ubiquitin ligase binding) coordinating with cell death pathways and chaperone activity; (ii) calcium-mediated neural plasticity (EF-hand domains, MAPK signaling); and (iii) integrated sensory/hormonal (olfactory transduction, secretory granules) and immune-fluid homeostasis modules (complement, aldosterone regulation).

Liver–heart analyses (Figure 3B,E,H) further confirmed metabolic tuning through targeted energy flux enzymes (enoyl-CoA hydratases, biotin carboxylases) and TCA cycle augmentation (fold enrichment: 1.56), enabling hypoxia-responsive ATP maximization. Critically, proteostatic adaptation manifested via ubiquitin–proteasome systems functionally linked to cell death control and proline isomerization. Cardiac specificity emerged through endocrine–immune crosstalk (thyroid/insulin secretion with NLR signaling) and conserved structural adjustments (spectrin mechanotransduction, HIF-1 stabilization of mitochondrial complexes, with a fold enrichment of 1.66 for this pathway).

Muscle–heart profiling (Figure 3C,F,I) uncovered opposing ATP production strategies: muscular glycolytic amplification (fructose-bisphosphate aldolase targeting, glycolysis) versus cardiac oxidative refinement (mitochondrial complex enrichment). KEGG analysis of this comparison further revealed robust enrichment of the TCA cycle (fold enrichment: 2.38) and HIF-1 signaling (fold enrichment: 2.69). Proteostasis was maintained through translational regulation (elongation factor domains coupled with RNA degradation), while electrophysiological precision was achieved via adrenergic signaling and M-band mechanotransduction. Purine biosynthesis and antioxidant domains (AhpC/TSA family) jointly preserved ATP synthase activity under contractile stress.

Lactylation mediates plateau survival through three convergent axes: metabolic channeling (tissue-specialized ATP generation), proteostatic vigilance (folding/degradation quality control), and structural–electrical calibration (excitation–contraction coupling optimization). This triad may support hepatic metabolic plasticity, cardiac endurance, and muscular power output observed in plateau-dwelling yaks, suggesting potential adaptive strategies.

### 3.4. Validation of Lactylation Sites and Tissue-Specific Associated Enzyme Expression in Yaks

**Figure 4:**
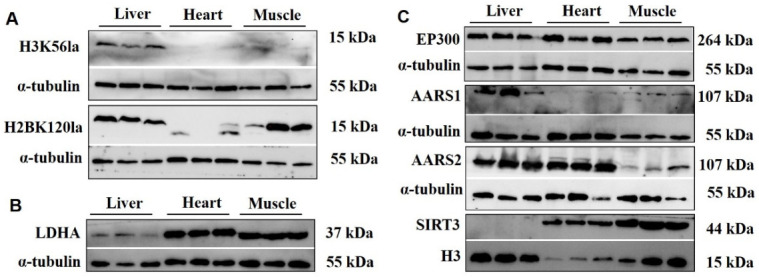
Tissue-specific patterns of lactate metabolism and lactylation regulators in yak. (**A**) Western blot validation of histone lactylation sites. (**B**) LDHA levels in yak tissues. (**C**) Expression of candidate lactylation regulators (EP300, AARS1, AARS2, SIRT3) across tissues.

Tissue-specific patterns of protein lactylation and their regulated enzymes were further investigated in yak. Histone analysis revealed significantly reduced H3K57la (L8I0H3_K57la) in liver compared to both muscle and heart (adjusted *p* < 0.05; Table 1), while H2BK121la (L8HRZ4_K121la) exhibited a distinct abundance pattern across all tissues (liver > muscle > heart, adjusted *p* < 0.05). Western blot analysis using antibodies targeting human homologous sites (H3K56la PTM-1421RM, H2BK120la PTM-1423; note: the corresponding yak sites are offset by +1 due to the absent of an N-terminal methionine, as validated by MS and the vendor) confirmed the tissue-specific differences identified by MS (Figure 4A). Investigation of the underlying metabolic mechanism demonstrated lower LDHA protein expression in liver tissue than in muscle or heart tissue (Figure 4B), suggesting attenuated lactate production. Analysis of putative lactylation regulators showed: no significant variation in EP300; tissue-specific elevation of AARS1 in the liver; differential AARS2 expression (liver/heart > muscle); and detectable SIRT3 in muscle and heart tissue but not in the liver (below detection limit; Figure 4C).

## 4. Discussion

Lactate, once considered a terminal metabolic waste product, is now recognized as a key signaling molecule that maintains physiological homeostasis across organ, tissue, and cellular levels via the lactate shuttle mechanism, and contributes to various disease processes [25]. A pivotal aspect of its signaling function is its role as a precursor for lysine lactylation, a novel post-translational modification whose abundance correlates with intracellular lactate levels and is regulated by specific acyltransferases and deacylases [8,9]. As an emerging field, evidence indicates that lactylation modulates gene expression, signal transduction, and metabolic reprogramming through actions on both histone and non-histone proteins, implicating it in diverse physiological and pathological pathways [11,12,13]. The yak, with its exceptional metabolic efficiency forged on the Qinghai–Tibetan Plateau, presents a compelling model to investigate the physiological roles of lactylation. While our earlier work demonstrated widespread lactylation involvement in energy metabolism across yak tissues [5], its quantitative, tissue-specific distribution and functional differentiation remained uncharacterized. This study systematically maps this landscape by integrating proteomic and lactylomic data from yak liver, muscle, and heart.

Our integrated proteomic and lactylomic analysis of yak tissues revealed a distinct “expression-modification decoupling” pattern in the protein abundance-versus-lactylation scatter plots (Figure 1A–C). This pattern, particularly the enrichment of proteins in Quadrant II (low abundance, high lactylation), suggests a potential mechanism for post-translational regulation independent of protein expression levels. For instance, LDHA exhibits lower protein expression but significantly higher lactylation in the liver compared to the muscle, implying that enhanced lactylation may compensate for reduced expression to enable precise lactate metabolic flux control. This pivotal role of the LDHA–lactate axis in fine-turning metabolic homeostasis is supported by interventional studies. Chlorogenic acid has been demonstrated to ameliorate high-fat-diet-induced metabolic dysfunction in skeletal muscle precisely by reducing lactate production and global protein lactylation, thereby highlighting the LDHA–lactate axis as a critical target for improving mitochondrial function [26]. Notably, in a subacute ruminal acidosis model in Holstein cows, elevated lactate in the mammary gland and plasma promotes histone lactylation, activating the TLR4/NF-κB pathway and inducing mastitis [27]. Nonetheless, the observed decoupling pattern likely represents a broader mechanism of metabolic fine-tuning, analogous to regulatory strategies seen in other PTMs such as succinylation [28] and glycosylation [29]. Collectively, our multi-omics data suggest a potential bidirectional feedback mechanism between protein abundance and lactylation that contributes to organismal physiological homeostasis.

In pairwise tissue comparisons, we identified 135 common differentially lactylated proteins (e.g., ENO1, HBB, and ALDOA), all of which exhibited pronounced tissue-specific divergence in both modification sites and intensity. This suggests that the same protein may undergo site-specific lactylation in response to distinct metabolic states across tissues. Notably, certain proteins, such as HBB, contained both up- and downregulated lactylation sites within the same comparative group, with modifications occurring at distinct residues. This phenomenon of bidirectional PTM is not unique to lactylation; for instance, in systemic lupus erythematosus, peripheral blood mononuclear cells exhibit both increased and decreased 2-hydroxyisobutyrylation at specific sites on 127 proteins compared to healthy controls [30]. Whether such bidirectional modifications represent an evolutionarily conserved fine-tuning strategy remains an open question.

Furthermore, lactylomic analysis revealed pronounced lactylation at K152 and K304 of SIRT3 in yak liver (Supplementary Figure S2K,L). Combined with its minimal protein abundance (as confirmed by Western blot), this suggests an inverse correlation between lactylation level and SIRT3 expression, implying that lactylation may trans-inhibit its deacetylase/delactylase activity. This model of a self-regulatory metabolic sensing mechanism is supported by non-enzymatic lactylation pathways; for example, lactoylglutathione (LGSH)—a conjugate formed between methylglyoxal, a glycolytic byproduct, and glutathione—can mediate lactyl group transfer and modify glycolytic enzymes, thereby feedback-regulating glycolytic flux [31]. Thus, our results raise the possibility that lactylation may constitute a similarly precise feedback regulatory system in yaks.

The modification levels of lactylation on proteins are influenced by a wide range of factors, from intracellular metabolic states to extracellular environmental cues [32]. Functional enrichment analysis revealed that tissue-specific lactylation in yaks is associated with multi-axis regulatory processes—including energy metabolism, protein homeostasis, and electromechanical coupling—which may be relevant to their adaptation to the plateau environment. Tissue-specific specializations were evident: the liver showed enrichment in mitochondrial energy production (TCA cycle, oxidative phosphorylation) and protein quality control; muscle displayed enrichment in glycolytic and thermogenic regulation; and the heart showed enrichment in oxidative metabolism, endocrine–immune crosstalk, and spectrin-based structural reinforcement. These lactylated proteins consistently localized to mitochondria, protein turnover complexes, and chaperone systems, suggesting systemic involvement in core cellular functions. Notably, low-altitude mice exposed to 3600 m for 40 days showed substantial ocular proteomic alterations involving complement activation, humoral immune responses, second-messenger signaling, leukocyte activation, and disrupted iron homeostasis, alongside significantly elevated ocular lactylation [33]. This comparative perspective is consistent with yaks having a multi-tissue, metabolically integrated profile, whereas mice mount a more acute adaptive response centered on immune–inflammatory and stress-signaling pathways. However, we acknowledge that these findings remain correlative; more definitive conclusions regarding altitude-driven lactylation changes would therefore require future studies involving either intra-species comparisons across different altitudes or controlled hypoxia experiments.

Distinct lactylation patterns across yak tissues target critical metabolic enzymes, potentially facilitating precise energy allocation and forming tissue-specific “metabolic boundaries” that optimize metabolic flux. For example, proteins ranging from oxygen-transporting HBB, glycolytic enzymes ENO1, ALDOA, and LDHA, to oxidative phosphorylation components ATP5C1 and ATP5H all exhibit tissue-specific differences in lactylation levels and site occupancy. This raises a fundamental question: is lactylation merely a passive marker of energy status, or an active driver regulating metabolic pathways? Accumulating evidence supports a dual role: on one hand, non-enzymatic lactylation mediated by LGSH exerts feedback regulation on glycolytic enzymes [31], underscoring its regulatory function; on the other hand, the dose-dependent relationship between lactylation levels and lactate concentration [8] supports its role as a metabolic status indicator. Crucially, site-specific engineering of lactylation into ALDOA has been shown to directly inhibit its enzymatic activity, revealing a lactylation-dependent feedback loop within glycolysis and affirming its active regulatory role beyond that of a passive lactate abundance marker [34]. This duality is also evident under pathological conditions such as tumor development. Under physiological conditions, lactate and lactylation help modulate immune suppression and inhibit tumor progression [35]. In contrast, during tumor progression, the enhanced Warburg effect leads to lactate accumulation in the tumor microenvironment, markedly elevating global lactylation levels, which in turn promotes malignant progression [35,36].

At the protein homeostasis level, lactylation in yaks may achieve precise regulation by modulating the ubiquitin–proteasome system and heat shock protein (HSP) functions. We speculate that lactylation might inhibit the degradation of specific metabolic enzymes by competing with ubiquitination for lysine residues or allosterically regulating E3 ligase activity—a mechanism analogous to TFEB K91 lactylation-enhanced autophagy [37]. This metabolic regulatory role may be a common, evolutionarily convergent function of lactylation. Studies in plants demonstrate that lactylation extensively targets nutrient reservoir proteins in rice grains and shows strong associations with central carbon metabolism and protein biosynthesis [38], suggesting it also plays a role in regulating protein synthesis. Furthermore, in yak muscle, although lactylated HSPs are significantly enriched, the functional consequences of this modification are likely complex and context-dependent. For instance, lactylation of Hsp60 can transform its role from a molecular chaperone to a driver of mitochondrial fission and apoptosis [39], suggesting that lactylation may induce functional reprogramming—rather than simply enhancing canonical functions—of HSPs to meet tissue-specific homeostatic demands. This hypothesis, however, requires further validation. In summary, the interplay between lactylation and ubiquitination is diverse and highly context-dependent [40], and the specific regulatory mechanisms operating across different yak tissues remain to be systematically elucidated.

This study further reveals that lactylation in yaks is associated with an immune–endocrine–antioxidant axis, potentially contributing to a tripartite defensive strategy integrating anti-inflammatory response, redox balance, and fluid homeostasis. Specifically, proteins with upregulated lactylation in yak liver—as compared to muscle—are concurrently enriched in complement and coagulation cascades, aldosterone/insulin signaling pathways, and AhpC/TSA antioxidant domains. Beyond these pathways, cytosolic AARS1 has been reported to lactylate cyclic GMP-AMP synthase (cGAS), thereby modulating innate immune responses [16], suggesting that lactylation may exert broader immunosuppressive effects beyond complement/NOD pathway suppression. In contrast to the canonical “hypoxia-inflammation vicious cycle” model often observed in human altitude sickness [41], yaks may achieve a physiologically “immunosilent” state through lactylation-mediated suppression of complement/NOD pathways, coupled with fine-tuned aldosterone-regulated water–electrolyte balance. Concurrently, the co-enrichment of AhpC/TSA antioxidant enzymes and purine nucleotide biosynthesis pathways establishes a molecular link between oxidative defense and ATP regeneration, ensuring dynamic equilibrium between energy supply and ROS clearance. Furthermore, significant enrichment of thyroid hormone signaling alongside aldosterone and insulin secretion pathways expands the regulatory dimension of the “heart as an endocrine organ” in yak [42]. These findings align closely with lactate’s established role as a core immunomodulatory molecule [43]. While these associations are intriguing, we recognize that enrichment analyses indicate pathway associations rather than demonstrating direct pathway activation or physiological functions; thus, these interpretations should be considered as generating hypotheses for future investigation.

Based on our quantitative PTM proteomics and Western blot analyses, we propose that distinct lactylation regulatory patterns exist across yak tissues. As shown in our results, lactylation levels vary considerably among tissues, with the liver exhibiting higher overall lactylation compared to muscle and heart. The tissue-specific expression of key lactate-metabolizing (LDHA) and lactylation-regulating enzymes (AARS1, AARS2, SIRT3) collectively underlies this tissue-type-specific regulation. For instance, high AARS1 expression in the liver may promote protein lactylation, while minimal SIRT3 expression likely impairs delactylation, together potentially leading to lactylation accumulation. Although EP300 expression is consistent across tissues, its established role as a lactyltransferase suggests a central network function, potentially regulated post-translationally, as EP300 knockdown markedly reduces global lactylation in other models [44]. It should also be noted that novel lactylation-regulating enzymes continue to be identified [9]. We acknowledge several methodological limitations in this study: the sample size (*n* = 3 per tissue) is modest, antibody-based enrichment of lactylated peptides may introduce potential biases, site-specific functional validation of individual lactylation events was not performed, and normalization procedures have inherent uncertainties. Despite these constraints, our data provide a valuable resource for understanding tissue-specific lactylation in a high-altitude mammal. The full regulatory landscape and upstream mechanisms governing tissue-specific lactylation in yaks require further elucidation via functional experiments and validation in larger cohorts.

## 5. Conclusions

The decoupling of lactylation from protein abundance suggests a transcription-independent mode of metabolic regulation, potentially enabling rapid tissue-specific responses. Notably, the lactylation of SIRT3—despite its near-undetectable protein level—implicates a self-perpetuating feedback loop that may lock in and sustain distinct modification landscapes. Together, these features allow lactylation to delineate sharp metabolic setpoints across the liver, muscle, and heart, which operates on a timescale that likely outpaces protein-level turnover and thus positions this modification as a key rapid-response interface for environmental stressors. We propose that this integrated post-translational rheostat coordinates tissue metabolic specialization and, through its rapid dynamics, may help circumvent the pathological hypoxia–inflammation cycle, thereby potentially contributing to high-altitude physiological resilience. This framework offers a mechanistic hypothesis in which dynamic modification circuits, rather than static protein abundance, may serve as key determinants of hypoxia tolerance—a model that now provides clear, testable avenues for future causal validation.

## Figures and Tables

**Table 1:** Differential histone lactylation in pairwise yak tissue comparisons.

Protein Accession	Protein Description	Liver vs. Muscle (Up)	Liver vs. Heart (Up)	Muscle vs. Heart (Up)
L8HRZ4	Histone H2B	K6, K47, K86, K109, K117, K121	K6, K47, K86, K109, K117, K121	K121
L8HXR5	Histone H4	K32, K78	K32, K78, K92	−
L8I0H3	Histone H3	K80, K123	K15, K24, K57, K80, K123	−
L8IJS0	Histone H1.0	K82, K85	K85	−
L8HPG7	Histone H2A	−	K96	−

## Data Availability

The mass spectrometry proteomics and lysine lactylome data have been deposited to the ProteomeXchange Consortium via the iProX partner repository with the dataset identifier PXD079220.

## Supplementary Materials

The following supporting information can be downloaded at https://www.mdpi.com/article/10.3390/ani16142228/s1, Table S1. Lactylated sites with regulation independent of protein expression in Quadrants II and IV; Table S2. Differentially lysine-lactylated sites and their corresponding proteins in pairwise comparisons of yak tissues (|log2FC| ≥ 0.585, adjusted *p* < 0.05); Table S3. Yak proteins harboring both up- and downregulated lactylated sites in specific tissue comparison groups; Figure S1. Quality control of proteomic and lactylomic data for yak liver, muscle, and heart. (A) PCA score plot of proteomic data; (B) PCA score plot of lactylomic data; (C) Pearson correlation heatmap among proteomic samples; (D) Pearson correlation heatmap among lactylomic samples.; Figure S2. MS/MS spectra of lysine-lactylated peptides derived from twelve lysine lactylation sites on seven representative target proteins. Red peaks correspond to N-terminal b-type fragment ions and blue peaks represent C-terminal y-type ions. The peptide sequence is displayed above each corresponding spectrum, with lactylated lysine labeled as LacK. Annotated peaks show matched fragment ions with experimental m/z values. X-axis: mass-to-charge ratio (m/z); Y-axis: fragment ion intensity. (A) ENO1_K126; (B) ENO1_K228; (C) HBB_K35; (D) HBB_K98; (E) ALDOA_K199; (F) ALDOA_K282; (G) LDHA_K184; (H) LDHA_K346; (I) ATP5C1_K83; (J) ATP5H_K117; (K) SIRT3_K152; (L) SIRT3_K304; Figure S3. PPI network analysis of the top 50 differentially lactylated proteins in pairwise tissue comparisons. (A) Liver vs. Muscle; (B) Liver vs. Heart; (C) Muscle vs. Heart; Figure S4. Functional classification of differentially lactylated proteins. (A–C) Subcellular localization; (D–F) COG/KOG analysis.

## Abbreviations

The following abbreviations are used in this manuscript:

AARS1	Alanyl-tRNA synthetase, cytoplasmic
AARS2	Alanyl-tRNA synthetase, mitochondrial
AhpC/TSA	Peroxiredoxins
ALDOA	Fructose-bisphosphate aldolase A
COG	Clusters of orthologous groups of proteins
DLD	Dihydrolipoyl dehydrogenase
ENO1	Enolase
EP300	Histone acetyltransferase p300
GO	Gene ontology
HBB	Hemoglobin subunit beta
HIF-1	Hypoxia-inducible factor 1
KEGG	Kyoto Encyclopedia of Genes and Genomes
Kla	Lysine lactylation
KOG	Eukaryotic orthologous groups of proteins
LC-MS/MS	Liquid chromatography-tandem mass spectrometry
LDHA	Lactate dehydrogenase A
MDH	Malate dehydrogenase
OXPHOS	Oxidative phosphorylation
PTM	Post-translational modification
ROS	Reactive oxygen species
SDH	Succinate dehydrogenase
SIRT3	Sirtuin 3
TCA	Tricarboxylic acid
